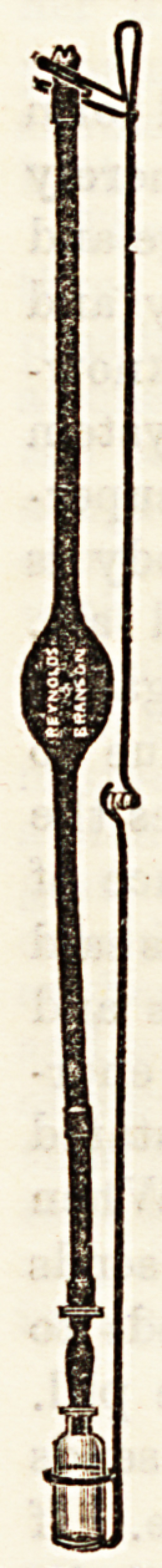# The Hospital Nursing Supplement

**Published:** 1895-05-04

**Authors:** 


### "The Hospital" Nursing Mirror.

Being the Extra Nursing Supplement of "The Hospital" Newspaper.

[Contributions for this Supplement should be addressed to the Editor, The Hospital, 428, Strand, London, W.C., and should have the word "Nursing" plainly written in left-hand top corner of the envelope.]

### News from the Nursing World.

### ROYAL INSPECTIONS.

Unfortunately red cloth and hired plants have come to be considered in the light of such indispensable accessories to Royal visits that even the plainest of institutions is temporarily disguised before its doors are opened to receive the honoured guests. Although the bare entrance hall may be transformed by these outer adornments, happily other parts of the structure preserve their identity, and give proof of the useful work in each department. Moreover, it is hardly yet sufficiently understood that scarlet baize and bunting do not blind the keen eyes of any Royal visitors. Our English princes and princesses are experienced inspectors, and to kindly interest add large personal knowledge of the constituents of efficient hospitals. It is therefore both as highly cultured practical men and women, and as gracious Royal patrons that they benefit the great charities where hearty welcome awaits the sons and daughters of our Queen.

### QUEEN VICTORIA'S JUBILEE INSTITUTE.

In concluding his summary of the work done by the Institute during the five years of its existence, the Rev. Arthur L. B. Peill, the president, says, "A Queen's nurse means, what it should mean, that those who hold that distinction, and wear the token of it in the badge and brassard, are rendering to the poor in their own homes the same skill and attention that others do to the rich, and are so fulfilling the Intention of Her Majesty in founding the institute. The nurses have contributed in no small degree to raise the Institute to the position it now holds."

### CERTIFICATED MIDWIVES.

Many nurses read with interest the abstract of Dr. Champneys' able address, delivered before the Obstetrical Society of London on the midwife question. He notes that the agitations got up of late years have been chiefly directed against "skilled" midwives who have gone through a due course of training followed by an examination, and are certified as competent to attend natural labours. In other words, as Dr. Champneys remarked, those who "have been taught how not to infect their patients, and when to call in the doctor." The history of English midwives was outlined in the course of this address, with the natural deduction that, being necessary persons, they should be educated and examined rather than left ignorant and septic. The interests of the poor found an eloquent advocate in Dr. Champneys, who defended their right to skilful attendants. As significant of the indifference of the public to the attacks made on the society, it is found that the candidates for the last examination were by no means below the average number. The Midwives' Registration Bill, which was read a first time in the House of Lords on April 30th, has been drawn up in accordance with the suggestions of the medical men of whom the "Midwives' Registration Association" is composed, and its clauses are published in *Nursing Notes* for May. It will probably come up for second reading in about ten days.

### HOLIDAYS WELL EARNED.

Under the patronage of the Duchess of Teck the annual meeting of the Factory Girls' Country Holiday Fund, of which Her Royal Highness is president, was held on April 29th in the Haberdashers' Hall. The rapid growth of the movement, started in 1888 by Miss Canney, has been remarkable, and last year 704 girls and women benefited by the fund. There was a large attendance the previous week at a drawing-room meeting at Mrs. Gilbert Samuel's, when various speakers dwelt on the advantages derived from an annual holiday by these hard-working poorly-paid girls. Miss Ravenhill, whose finished elocution made all that she said doubly attractive, spoke on the hygienic view of the subject. In pointing out the obvious economy of preserving the health of working women by country visits, and, even by the conventional summer excursions, she did not lose sight of the wealth of new ideas thus introduced into the monotonous lives of keen, active-minded factory hands. The National Health Society may well congratulate itself on ranking Miss Ravenhill amongst its lecturers, for she is not only a remarkably fluent speaker, but she has that sound knowledge of her subject which wins the instinctive confidence of an audience. The factory girls should benefit substantially by the publicity given to their needs and their deserts.

### NORTH LONDON HOSPITAL FOR CONSUMPTION.

The Hospital for Consumption at Mount Vernon is such an imposing-looking structure that one is surprised to find it only accommodates sixty patients. The wards are lofty, with wide windows, and the galleries answer the purpose of day rooms. The patients are admitted for six weeks only, and they must leave their pleasant surroundings regretfully. A fine room, recently added, answers for a refectory for both male and female patients, the matron presiding, and seeing that each fanciful appetite is suited. She is excusably proud of this new addition, which is also used for entertainments for the patients. The rooms of the nursing staff are pleasant and airy, but they are placed in undesirably close proximity to those of the servants. The kitchen, on the same floor, is a bright one, and conveniently near to the nurses' dining-room.

### WORK FOR WOMEN.

Some interesting facts were placed before the audience which assembled at St. Leonards on April 26th to listen to a lecture by Miss Goodrich Freer on "Women as Horticulturists." She spoke with approval of the free scholarships offered by the County Councils of Berks, Kent, and Essex, of which several students at the Swanley Horticultural College have already availed themselves. Beekeeping, poultry rearing, dairy work, table decorations, bouquet making, and every branch of practical and theoretical gardening are included in the course given at the college. Miss Freer said that the demand for qualified women gardeners at present exceeded the supply. It therefore seems as if this womanly industry were not yet in danger of the overcrowding unfortunately characteristic of many other callings. Sound health, energy, patience, and perseverance are required in a successful gardener, and women must face the necessity for making their training as thorough as possible and sparing no pains in obtaining a complete preparation for an interesting and profitable career.

### A LECTURE FOR NURSES.

At the Trained Nurses' Club in Buckingham Street a crowded audience assembled on April 26th to hear Dr. Herman on "The Consequences of Mismanaged Labour." The closely-packed groups of nurses listened with keen interest to the lecturer's instructions, comments, and warnings, for Dr. Herman laid much stress on the responsibilities of the trained nurse towards doctor and patients. He also emphasized the necessity for bestowing proper care on the mother during the whole lying-in period. The Buckingham Street club is deservedly popular, and the sitting-room, well stocked with periodicals, forms a pleasant central rendezvous for the members.

### A NAMELESS NURSE.

Again we have to reproach a correspondent for omitting her name and address. The letter signed "A Nurse," giving a warning which might be of value to others, is rendered useless by this omission. We have to remind all our friends that communications which bear no name or address cannot receive attention. Perhaps "A Nurse" will remedy her omission at once, if she really desires to have an opinion on her letter.

### WOMEN LECTURERS.

The dignity of women lecturers was not advanced by Miss Kenealy's recent address on this branch of work. Much stress was laid on comic and frivolous experiences, the sober realities of health teaching receiving but secondary consideration. The County Council modes of appointing lecturers were as severely criticised as the lukewarmness of the clergy in matters of technical education. With Miss Kenealy's condemnation of noisy chatelaines and showy uniforms all good nurses agree, whilst none deny the popularity which attaches to a quiet and neat nurse's dress on women entitled to wear it.

### NURSES AT DUNDEE.

The results of the annual examination held at the Dundee Royal Infirmary for the nurses attending the course of lectures delivered by the Medical Superintendent are considered most satisfactory. The examination took place on April 6th, and, according to custom, the House Committee awarded their three annual prizes. The third year first prize was awarded to Nurse R. Hunter, and the second year first prize to Nurse H. Henderson, whilst the winner of the first year first prize was Nurse McIntosh. Nurse Henderson was placed first on the list with 113 marks out of a possible 120.

### NURSES FOR NORTHERN INFIRMARIES.

The Northern Workhouse Nursing Association requires more money, for it expends some £400 per annum and receives only £250 in subscriptions. The Northern Association undertakes 225 unions, each having sick wards or its own hospital; and it trains nurses specially for this work, providing a course of midwifery instruction for candidates selected by the committee. The Duchess of Westminster is the president of the association, and the committee is an influential one, the names on the list of vice-presidents sufficiently guaranteeing the worth of a society which assuredly deserves wide support. In the course of last year 42 nurses were supplied in response to the request of boards of guardians in northern districts, and the association has 21 probationers training for future work.

### IS BOMBAY PHILANTHROPIC?

The present financial difficulties of the nursing department of St. George's Hospital, Bombay, have led to the disclosure in the *Times of India* of a somewhat remarkable state of things. It appears that from twenty-four to thirty-one nurses are employed for day and night duty at this hospital, and the average cost of the establishment is Rs.1,500 per mensem, and that while the Government and the Endowment Fund together contribute Rs.990 per mensem, the balance, some Rs.500 is raised by the earnings of the forty private nurses attached to the hospital. Until recently the private nurses' earnings have sufficed for the maintenance of the hospital nurses, but the balance at the bank is at last overdrawn. Moreover, for two years nothing has been paid into the Provident Fund, established to ensure gratuities for nurses on their retirement. The All Saints' Sisterhood took the nursing in hand in 1884, and in 1889 by request of the sister superior an influential committee was appointed to receive and manage the funds. The honorary secretary speaks of the institution as having been hitherto "self-supporting," but that expression hardly seems descriptive of a system by which the earnings of one class of nurses are appropriated for the support of a public hospital. This plan having absorbed for the last two years the money which should have gone to their Provident Fund, after doing private nursing in India these broken down or worn-out nurses cannot even count upon the so-called gratuity to which they are assuredly entitled. If Bombay cannot do better than this, English nurses had better stay away. Large fees are hardly earned in an Indian climate, and when these all go to the support of other workers, the future outlook of both classes of nurses is sufficiently dreary. If the hospital were properly supported by the Europeans, who chiefly benefit by it, private nurses could provide for their own future even if they received more moderate fees than those which now apparently place their services beyond the reach of any but the most wealthy members of the Anglo-Indian community.

### SHORT ITEMS.

We understand that a report is current that Miss Philippa Hicks may yet be induced to reconsider her intention to withdraw from the superintendence of the nurses' co-operation.

### Elementary Anatomy and Surgery for Nurses.

By W. McADAM ECCLES, M.B., M.S., F.R.C.S., Lecturer to Nurses, West London Hospital, &c.

### XV.—THE CIRCULATORY SYSTEM.

IN order that the nutriment which has been prepared by the digestive system, and the air which has been received into the lungs may be distributed throughout the body, a fluid called blood is circulated through vessels which permeate nearly every tissue of the body, and the force required to propel this fluid is produced by the muscular organ known as the heart.

### THE BLOOD.

The blood itself is made up of two chief constituents:—(1) the liquid portion—the liquor sanguinis, or the plasma; (2) the solid particles, floating in the fluid—the corpuscles, which are of two kinds, (*a*) the red, the most numerous, are

FIG. 23.—BLOOD CORPUSCLES.

circular biconcave discs; (*b*) the white, which are true cells, occur in the proportion of one to five hundred red. (See Fig. 23.) When blood is withdrawn from a blood vessel it usually speedily clots, becoming a semi-solid mass from which serum, or the liquid part, afterwards drains away.

### THE BLOOD VESSELS.

The distribution of the blood is brought about by:—(1) the arteries, (2) the capillaries, (3) the veins, and (4) the heart; and these are grouped into three divisions, (*a*) the systemic

FIG. 24.—THE SYSTEMIC, PULMONARY, AND PORTAL CIRCULATIONS.

circulation, (*b*) the pulmonary circulation, and (*c*) the portal circulation. (See Fig. 24.)

The arteries are the vessels which convey blood from the heart to the tissues. They are tubes with muscular walls, and after death are usually found empty, hence the ancients thought they contained air, and so named them arteries.

The arteries divide and sub-divide until very small vessels are formed which are termed capillaries, the total sectional area of which is many times greater than that of the arteries.

The minute capillaries gradually unite with one another to form larger and still larger tubes called veins, the great characteristics of which are that their walls are much thinner than those of arteries, having but little muscular tissue in them, and that they contain more or less perfect valves in many parts.

### THE HEART.

The heart is a hollow muscular organ situated in the thorax in the middle line, but extending considerably to the left, and lying just above the diaphragm. It is surrounded by a membrane called the pericardium, which is composed of two layers, one investing closely the heart itself, and the other enveloping it loosely. Both surfaces in contact are smooth, and so facilitate the movement of the heart as the peritoneum does the movements of the abdominal viscera. The organ is in the main composed of a peculiar kind of muscular tissue which is found nowhere else in the body. It weighs about nine ounces in the adult. Its shape is somewhat conical, the base being uppermost, and the apex downwards and to the left, the beat of which when the heart contracts can normally be felt in the fifth left inter-

FIG. 25.—THE HUMAN HEART.

*ra*, Right auricle; *rv*, right ventricle; *la*, left auricle; *lv*, left ventricle; *ao*, aorta; *pa*, pulmonary artery; *vc*, vena cava superior; *pv* pulmonary veins.

costal space about three inches from the left border of the sternum. The heart contains four cavities, two on either side, called respectively an auricle and a ventricle, right or left as the case may be. Into the auricles empty the veins, from the ventricles start the arteries. Each of these cavities requires some description in order that the function of the heart may be understood. (1) The right auricle is formed of a comparatively thin wall with but little muscular tissue. It has an appendage shaped something like a dog's ear, hence the name of the cavity. Into this auricle open two great veins, the superior and inferior vena cava, the former collecting blood from the head, neck, arms, and thorax, the latter returning blood from the abdomen and the lower extremities. The veins of the heart itself also open into this chamber. (See Fig. 25.) (2) The right ventricle contains much more muscular tissue in its wall than does the auricle, with which it communicates by the right auriculo-ventricular opening guarded by a valve with three cusps, and therefore called the tricuspid valve. The margins of the cusps have fine cords attached to them coming from muscular papillæ on the wall of the ventricle. This valve allows blood to pass from the auricle to the ventricle but not in an opposite direction. In transverse section the cavity of the ventricle will be seen to be semi-lunar. Each ventricle will contain about four ounces of blood. Coming off from the right ventricle at the upper and left-hand corner is the pulmonary artery, which conveys venous or impure blood from the heart to the lungs, there to be aërated. The commencement of this artery is guarded by three semi-lunar valves.

### Nursing in America.—The Convention of Superintendents and Members of the Association.

### THE THREE YEARS' COURSE OF TRAINING IN CONNECTION WITH THE EIGHT HOURS SYSTEM.

By Mrs. Hunter Robb (Isabel Hampton), late Superintendent of Nurses at Johns Hopkins Hospital.

(*Concluded from page* xxvi.)

Further expense could be saved by having only one responsible head nurse, under the superintendent of the hospital, for the domestic management. In fact, it is only by such an arrangement that the third year's training could be made as practical as it should be. This position should be occupied by the superintendent of nurses and principal of the training school, so that besides the responsibility of the work of the nurses in the wards, she should have the care of the nurses' home, the linen-room, the laundry, and the buying for the hospital. Her staff should consist of a graduate head nurse in each ward, one for the nurses' home, and one for the laundry and linen-room, and one for the office. Their assistants in all these departments should be drawn from the pupil nurses of the third year; the head night nurse might also be a third-year nurse. The division of the practical work during the three years might be somewhat as follows:—For the first two years: Four months in the medical wards; four months in the surgical wards; three months in the gynæcological wards; one month in obstetrics; two months in the children's wards; three months in the private wards; two months in the operating-room; one month in the diet school; one month in the dispensary; one month on special duty; and one month on vacation. For the third year: Two months in obstetrics; four months as assistant in superintendent's office; three months as assistant in laundry and linen-room; and three months as assistant in nurses' home. During the six months in the superintendent's office, the assistants preparing for hospital positions would be expected to give a certain amount of class teaching to pupils of the first and second years. Nurses preparing for private duty should spend part of their third year in the wards, but all should serve their time in the linen-room and in the performance of the housekeeping duties at the home.

The first two years' teaching would consist of classes and lectures covering about the same ground as at present. Class instruction could be given twice instead of once a week, and since the pupils would have more time, and the instructors would be more numerous, the various subjects could be dealt with much more thoroughly than with our present system. For third year students class instruction could be given once, or perhaps twice, a week. The first four months of the first year could be devoted to class instruction on practical nursing and materia medica only, the second four months to human anatomy and physiology. At the end of the first year examinations might be held upon: 1. Practical nursing; 2. Materia medica; 3. Anatomy and physiology; 4. Diet. At the end of the second year: 1. Children; 2. Medical nursing, including massage, the examination of urine, and hygiene; 3. Surgical nursing, including the duties of the operating-room and the nurse's duty in emergencies; 4. Gynæcological and obstetrical nursing. The third year examination should include: 1. Methods to be adopted in class teaching; 2. Administrative duties of superintendents of training schools; 3. Practical care of the wards, the nurses' home, linen-room, and laundry; 4. Hospital buying and supplies; 5. Private nursing.

I need not say that the above is only a suggestive sketch for the third year teaching; I have only tried to indicate the leading points. It will remain for the association to draw up a schedule which with certain modifications can be made applicable to all training schools.

Among other things it will be their duty to decide upon the necessary qualifications of applicants, the standards of examination, the term of probation, and to provide for other emergencies. My object at present is to put before you the leading points; when these are settled the rest can, I think, be comparatively easily arranged.

The daily division of work for the eight-hour system could be made to work very nicely and interfere little, if any, with the present hours for meals by taking as a basis the hours four and four for some of the nurses, and six and two for the remainder. For instance, in a ward of 30 patients with six nurses, supposing the entire staff comes on at seven a.m.,

| | | | Hours' work. |
|---|---|---|---|
| Two are sent off at 11 a.m. ... ... | 1st din. | (2) | 4 + 4 |
| Same two return from 7 until 11 p.m. | 1st sup. | (2) | |
| Two off from 11 until 1 p.m.... ... | 1st din. | (2) | 4 + 4 |
| With same two on from 1 until 5 p.m. | 1st sup. | (2) | |
| Two on from 7 until 1 p.m. ... ... | 2nd din. | (2) | 6 + 2 |
| Same two on from 5 until 7 p.m. ... | 2nd sup. | (2) | |

The night nurse from eleven p.m. until seven a.m. In this way either the hours seven a.m. until eleven p.m. may be taken, or hours from half-past six a.m. to half-past ten p.m., or hours from six a.m. to ten p.m.

With this plan the nurses' classes and lectures could very well be arranged, and one, two, or more nurses could be sent off at once, according to the condition of the wards. In this way the full staff could be on during the busy hours of the morning, and there would always be two nurses in the ward during meal time. The hours of the head nurse and of her first assistant or senior, who would always be a third year nurse, should be so arranged that one or the other should be in the ward at all times during the day, and that both should never be absent at the same time.

These are some of the conditions under which I think the three years' course could be successfully adopted. It would possibly not be advisable to try to alter the present condition at one stroke, but to make the changes gradually, so that in the course of the next five years the new system could be adopted in all our good schools. Another consideration in connection with the subject is the co-operation of the larger with the smaller hospitals, but this I must leave to be discussed at some other time.

In conclusion, I would suggest that a chairman and committee be appointed from the present convention to draw up a plan based somewhat upon the lines which have been suggested in this paper. That this plan, after having been duly considered, should be forwarded by the committee to the authorities of the various hospitals for their consideration and approval, and that the committee should ask that a trial of such a scheme may be permitted for a certain length of time in certain hospitals selected for that purpose in order that it may be thoroughly tested, after which such action may be taken as the results of such trials would seem to indicate.

### A Nurse at Aden.

News comes from Aden of the temporary detention there in quarantine of Miss Wildman, who was formerly superintendent of the Ramsay Hospital, Naini Tal. Since she resigned that appointment last autumn Miss Wildman has been nursing in Calcutta. She was returning to England in the ss. "Coromandel," and disembarked at Aden to nurse a lady, who afterwards died of small-pox. Instead of pursuing her voyage, Miss Wildman, therefore, found herself constrained to remain for awhile in the European General Hospital at Aden, the P. and O. refusing to carry her on until a due period of quarantine had been observed. Miss Wildman's unselfish devotion has gained for her general respect.

### Letters from Upper Burma.

By Mrs. Ernest Hart.

### I.—BURMESE DOCTORS, MEDICINE, SPELLS, AND CHARMS.

In Burma good and bad spirits, who take an active part in the affairs of life, are firmly believed in; hence witchcraft is a living power, and charms and spells are studied, and are relied upon with the utmost faith. There is scarcely a Burman who does not bear somewhere about his body little rods and discs of silver, which are inserted under the skin to protect him from sword cuts or bullet wounds. Various figures and devices are also tattooed on the body, and are said to give absolute protection against snake bite. On an English doctor expressing in my presence incredulity on this point to a Burman, who had had some medical education in an English hospital, the Burman replied with great earnestness, "Oh, but, indeed, it will protect you, and if you doubt it we will tattoo you on the wrist, and next week we will bring a poisonous snake and it shall bite you, and you will see that no harm will result." It is needless to say that the English doctor declined to have the experiment tried. He had not the robust faith of a Burman who had a certain device tattooed on the chest, which was said to give absolute protection against drowning. Directly the operation was over, the man, who was a fisherman, insisted upon having the charm put to the test. He induced his friends to accompany him to his boat, and to display still more the great value of the charm, he was bound hand and foot. His friends rowed into mid-stream, and at his request they threw him overboard. To their great surprise and dismay he sank and was drowned. When they were afterwards brought to trial for manslaughter the serious legal defence was that there was some error in preparing the charm, or possibly the spirit of the river had been displeased, hence the charm did not act as it certainly would have done had everything been carried out correctly.

The Burmese doctor need go through no prescribed form of education, nor does he take any diploma. It is merely necessary for a man to assert he has medical knowledge and skill, and a credulous public believe him. Of anatomy and physiology the Burmese doctor is ignorant, and his knowledge of drugs is less than empirical; in fact, his system of medicine is a surprising mixture of ignorance and superstition. Burmese doctors consider that the human body is composed of four elements—earth, water, fire, and air. Earth constitutes the flesh, bone, hair, and internal organs; water constitutes the blood, secretions and fat; it is due to the fire element that man eats and drinks; air produces the six kinds of wind. It is the disturbance of the balance of these which causes illness. The circulation of the blood is said to be due to the wind driving on the blood. The pills and potions with which a patient is drugged consist of an enormous number of ingredients. A certain green powder, stated to be infallible in its results, contains 160 ingredients. When a man is ill, he becomes much excited and alarmed, and sends for several Burmese doctors. The first comer proceeds to examine his patient's horoscope and to prescribe some pill. If the sick man is not better in a quarter of an hour he sends for another doctor, who prescribes a different medicine. If recovery is not immediate, other doctors are sent for till the patient has been seen and dosed by all the doctors in the neighbourhood. He may succumb to the severity of the treatment, or he may recover in spite of the treatment. In the country districts, if the disease does not yield to drugs, it is said to be due to possession by an evil spirit. Incantations are then resorted to to expel the evil spirit; if he prove rebellious the patient is often submitted to the severest treatment, being beaten with sticks till he shrieks and groans. In surgical emergencies the Burmese doctor is powerless. He does not know how to set a broken limb nor how to stop bleeding; but he is said to be highly successful in preparing love potions and philtres. Though so ignorant of anatomy, the Burmans are, nevertheless, good sham pooers. These are nearly always women, who are acknowledged on all sides to be the most intelligent half of the Burmese race. The shampooer's knowledge of the muscles and tendons of the body is surprising, considering that she has never had the opportunity of studying anatomy. Bewitchings are believed in so firmly that when incantations and beatings have proved unavailing the complainant will sometimes seek the protection of the courts against the witch; but to the disappointment of the Burman the English magistrate declares the case to be beyond his jurisdiction. When cholera attacks a village the inhabitants make the most frightful noise and din by beating gongs and tom-toms and striking tin kettles, &c., with the object of frightening away the demon of the disease. The treatment of a woman after childbirth is remarkable. In order to get rid of the humours which are supposed to be in her she is submitted to a fiery ordeal whatever may be the heat of the weather. A large fire is lit in the room, blankets are heaped upon the patient, hot bricks are placed in the bed close to her body, and she is made to drink a variety of decoctions. At the end of a week she is allowed to take a cold bath. This treatment does not kill, but it ages her.

### The Nursing World.

So far as the evidence in our possession goes, and it is somewhat extensive, there must be quite 25,000 women engaged in some branch of nursing within the confines of the United Kingdom at present. If the words "nursing world" have any meaning, they must necessarily include these 25,000 women, if they do not indeed cover, as we suppose they do, everybody engaged in nursing throughout the world. In such circumstances it is intensely funny to notice that an insignificant meeting of some fifty individuals at the outside is described in a contemporary as a "large, interesting, and very representative gathering of the nursing world." The Matrons' Council has never attracted hospital matrons in any proper meaning of that term, and to describe it or its meetings as "large, interesting, and representative of the nursing world" is to reduce the whole thing to a farce, and cannot fail to cover the Matrons' Council with ridicule. We commend "Æsop's Fables" to the careful study of those responsible for the statement, and would specially call their attention to the fable of the ass in the lion's skin.

### Invalid Children's Aid Association.

This association, which has offices at 18, Buckingham Street, Strand, was originally established to look after delicate and crippled children in their homes. It has steadily grown and prospered, and it endeavours, amongst other good plans, to provide every little invalid brought to the notice of the hon. secretary with a friend of his own. These lady visitors find out the needs of the children, and the association supplies the perambulator or garment wanted. It lends invalid carriages, and pays for long sojourns in convalescent and other homes. The seventh annual meeting will be held on Friday, May 10th, at 24, Park Lane, by the kind permission of Lord Brassey, at four p.m.

### French Schools for Trained Nurses: Their Origin and Organisation.

(*Continued from page* cxxxiii, *Vol. XVII.*)

III.—THE HOSPITAL NURSING STAFF.

The hospital nursing staff both male and female, is chosen by the hospital directors, of which there is one to every hospital, who selects his own staff. Neither male nor female nurses are required to have a nurse's certificate in order to obtain the position of hospital nurse, and in consequence of this very serious omission, which will probably soon be remedied, many hospital directors oblige both male and female candidates for the post of hospital nurse to undergo a preliminary examination, consisting of an easy dictation and a few easy sums. This test is regarded as an indication that the candidates are not absolutely ignorant and incapable. Dr. Bourneville, the editor of the *Progrés Médical*, and the energetic organizer of the Paris nursing schools, clearly perceives that hospital nurses should be called upon to furnish a certificate of *enseignement primaire* (elementary education) before becoming a hospital nurse. In an address given by him on July 30th, 1892, at the nursing school attached to the Salpetrière Hospital, Dr. Bourneville stated that with regard to the general instruction of hospital nurses, apart from special knowledge, his ideal nursing staff is one which does not include among the subordinates any one, male or female, who has not gained at least the *certificat d'études*.

In order to organise this ideal staff dreamed of by Dr. Bourneville, the hospital administration must recruit the nursing staff from men and women who have this certificate, and the young members of the subordinate staff of both sexes should be urged to regularly attend the primary school classes connected with the professional Bicêtre and Salpetrière schools, in order to obtain the required *certificat d'études.*

A year before Dr. Bourneville delivered this address at the Bicêtre school, he stated that the administration of the *Assistance Publique* had drawn up a regulation to the effect that every hospital nurse desiring to fill the post of *suppléant* or *suppleante* will be called upon to prove that besides the diploma and guarantees of good morals and good conduct, the candidate had received an elementary education. Such proof, according to the regulation, to be an examination in reading, writing, and arithmetic. Dr. Bourneville further pointed out that an examination of this kind is required from nurses not seeking promotion, but on entering a hospital as nurse. He hopes that the day is nigh when the *Assistance Publique* will exact from all candidates for places as hospital nurses a similar examination before entering on their duties.

At a recent interview with the capable organiser of the Paris nursing schools, Dr. Bourneville very kindly furnished the writer with a mass of interesting details. According to him there are at present great difficulties in the way of the requirement that hospital nurses should be recruited from men and women who have had a fair elementary education, inasmuch as they are badly paid, the average wages being £1 a month. The hospital directors are thus reduced to the necessity of taking what they can get rather than being able to choose from desirable candidates.

Dr. Bourneville in an address delivered at the training school of the Pitié Hospital on July 24th, 1894, remarked that the night-nursing ought to be reformed. In a great many hospitals it is the custom to require beginners to undertake to sit up at night so as to take charge of the patients. This is a great responsibility to undertake at the onset of a nurse's career, and frightens away a great many women who might become efficient and humane nurses.

It has already been stated that the wages of the nurses in Paris hospitals are inadequate to command the services of men and women sufficiently well educated for their duties.

We here subjoin a list of wages paid to the different grades of the nursing staff. Some members are paid entirely in money, and others partly by food, lodging, firing, &c.

Members of the Paris Hospital Nursing Staff who Receive Cash Wages.

Male and Female Superintendents, and Male and Female Sub-Superintendents. — Surveillants and surveillantes, 2,100 francs (£84) per annum; sous (sub) surveillants and surveillantes, 1,800 frans (£74) per annum.

Head Nurses.—Suppléants and suppleantes, infirmiers and infirmières, 1,440 francs (£57 12s.) per annum.

Nurses.—First class male and female nurses, 1,350 francs (£54) per annum; second class male and female nurses, 1,250 francs (50) per annum.

Members of the Paris Hospital Nursing Staff who are paid partly in Money and are Lodged, Fed, Clothed, and allowed Firing, Lights, and Washing.

Superintendents.—First class surveillants and surveillantes, 800 francs (£32) per annum; second class surveillants and surveillantes, 700 francs (£28) per annum.

Sub-Superintendents.—First class sous-surveillants and surveillantes, 600 francs (£24) per annum; second class ditto, 500 francs (£20) per annum; suppleants and suppleantes, 425 francs (£17) per annum.

Head Nurses.—Infirmiers and infirmières, 400 francs (£16) per annum.

Nurses.—First class infirmiers and infirmières, 380 francs (£15 4s. 2d.) per annum; second class infirmiers and infirmières, 350 francs (£14) per annum.

(*To be continued.*)

### Novelties for Nurses.

NEW ENEMA RACK.

The practical little contrivance introduced by Messrs. Reynolds and Branson will be found a real boon to nurses. The preservation of enema syringes is always an important matter, and this rack will be found to meet a want in the sick room. The little bottle to receive the drippings, as shown in the illustration, secures cleanliness and saves the nurse trouble. This most useful little article is wonderfully cheap, and as there is no reason why it should ever get out of order, it should be regarded as an indispensable accompaniment of the enema syringe. The rack can be obtained from Messrs. Reynolds and Branson, Briggate, Leeds.

A USEFUL TRAVELLING COMPANION.

Many persons have found that the generally-used "sponge bag" is apt to play them false, to the detriment of the contents of the travelling trunk. Messrs. Fisher are offering a most practical substitute in a tin sponge-box, which, whilst it is moisture proof, is so arranged as to admit of the ventilation of the contents and thus is usefully devoid of objections. The sponge box can be obtained from Messrs. Fisher, 188, Strand.

### Nursing in India.

Miss Pearse, late of Great Ormond Street Hospital, has been promoted to the rank of Deputy Superintendent at Peshawer, vice Miss Harris, resigned.

### The Great Lahore Durbar of 1894.

BY AN ENGLISH NURSE.

WITH great alacrity I accepted an invitation to join a ladies' camp attached to one of the Hussars regiments to witness the Durbar. I set off for Lahore with my man-servant, dog, my sais, pony and trap, on my eight-hour railway journey. My faithful Abdool found me a first-class carriage with one occupant, and put in my baggage, the latter filling up a considerable portion of the carriage. Just as we were starting I discovered my only companion would be a native gentleman, and as it is not considered the thing for a lady to travel in India with a native gentleman, I had to apologise for intruding, and leaving my baggage to his care, I entered the next carriage with a party of ladies bound for the Durbar. I afterwards recognised my native gentleman in the Rajahs' procession in the suite of the Rajah of N. Most of the ladies were bound for the same camp and travelling in glorious uncertainty as to where to go on arrival. The regiment were camped in a place it was out of the question for us to go to—about seven miles from Lahore and five miles from everywhere else. At Lahore the regimental drag met us, and as most of us had brought or sent carriages, we were well provided for, and fortune favoured us, for we had camping ground on the Mall, opposite Government House and near to everything. On reaching our camp, we unpacked our things, and began to get the tents in order; we bought matting for the floors from an itinerant Chick-wallah who was waiting.

One of the majors had gone on before with his wife, and she had a charming tea waiting for us in half of her tent, which she had done up as a drawing-room, and under these circumstances camp life promised to be charming. After tea, numbers of people came to call; we found there were two other ladies' camps in the same compound. Towards eight we began to feel hungry, and the junior major gave orders for ekkas to go out to the regimental camp for necessaries.

Knowing our commissariat was with the Hussars, and all arrangements for the mess miles away, our hearts became sad. Some retired to the senior major's wife's tent and regaled on biscuits and soda water. Between nine and ten p.m. dinner was announced and we mustered cold and hungry. Lights were borrowed from various tents, also chairs and tin-plates and tumblers from the tiffen baskets.

The floor of the mess tent represented the waves of the sea in point of irregularity, and we had some difficulty in moving about, and, as most of our chairs were low wicker ones, our heads were not very high above the table.

Soon after dinner we all went to bed, slept well, and rose at seven the next morning. The younger members of the party went for a drive and saw all the preparations for the reception of the Viceroy. The roads were lined with soldiers for miles, and as six of the regiments were well known to me, I recognised many old friends down the lines. After breakfast we went to Government House to see the procession pass. An A.D.C. kindly invited us to sit in one of General Lord Frankfort's carriages. On our right were drawn up the generals and staff; there were seventeen generals there altogether.

The escort of the Viceroy consisted of A Battery, R.H.A., the 11th Hussars, the Seaforth Highlanders, and the Viceroy's bodyguard. The latter are all picked men, over six feet high, and carry red and white flags on their lances. The traffic on the railway was so great that the Viceroy's carriages and horses were delayed and never arrived for the durbar at all, and he had to ride in a hired carriage. The levée was held the same evening. The ladies spent the day in calling at the Lieutenant-Governor's, the Viceroy's, the Commander-in-Chief's, the General commanding the station, on all the public officials, and on many private friends. At most places we only wrote our names in the books and chatted with the aides-de-camp. Next day we went early to Lahore, where the shops are lovely and the houses palatial, the streets or roads wide and planted with trees, chrysanthemums growing everywhere in the wildest profusion. After breakfast we hurried to the gate of the compound to see the rajahs pass on their way to the Viceroy's camp to call on his Excellency. Then we saw what Oriental splendour really is! The Maharajah of Patiala rode in a silver carriage ornamented with gold; he wore the jewels that were bought for him from the Empress Eugénie. He, like the other rajahs, was dressed in the richest of brocades. His troops, *i.e.*, his bodyguard, preceded and followed him. They were Hussars, in green uniform and gold lace. In the afternoon we went to the races, at which the attendance was at first rather small, for there had been a rehearsal of the review in the morning, and the officers could not get down in time.

Next day there were Highland games, very good and home-like, and the resemblance was further enhanced by heavy rain at intervals. The three Highland regiments, the Argyll and Sutherlands, the Gordons, and the Seaforths, acted hosts, and the display in the refreshment tent of regimental plate was grand.

The rain ceased as we returned to the camp, and found our mess more comfortable—glass and crockery and other marks of civilisation.

After dinner we went to an "At Home" at the Commander-in-Chief's, at which hundreds of guests were present. Large bonfires were blazing on the lawn to keep us warm, everyone was in full dress uniform or smart cloaks, and the rajahs' brocades glistened in the firelight. There was a tattoo and torch-light procession through the camp. As it was St. Andrew's Eve, the massed bands of the Highlanders played fine hymn tunes quite unexpectedly, and as they came through the night air, they seemed to still the gaiety that had pervaded the durbar week and to bring solemn thoughts. It is impossible to describe the beauty and comfort of the Viceroy's and Commander-in-Chief's camps, they were simply perfect.

The glories of that grand review next day were beyond description. On the plain of Mian-Nui a huge army was planted out consisting of forty regiments. The picturesque native troops consisted of wild looking Pattians, Sikhs with their long beards turned round the back of their ears, and little Gurrkhas, with black monkey-like faces and black uniforms. A crowd of generals were mounted in front of the saluting point. Sir George White, the Commander-in-Chief, headed his old regiment, the Gordon Highlanders. Clouds of dust like columns of smoke veiled the troops from us every now and then. Streams of gaily-dressed people on drags, coaches, gharries, and every sort of vehicle were there. But amid all this excitement there came a lull, that awful feeling that 'something' had happened. Though I was dressed in ordinary attire, I was recognised, and told in a hurried whisper I was wanted. The linch-pin had shot out from the fore wheel of one of the guns, and as the battery came galloping past the grand stand the wheel rolled off, and over came the carriage, crushing a horse and man beneath it, and another man had fallen on his head. Promptly the gallop past was stopped, and the poor driver, with crushed-in chest, was extricated. Keen on duty, and regardless of pain, he tried to mount three times to save the others, but he had to be carried off. Lord Harris, the Governor of Bombay, was so struck by the pluck of the poor gunner, which he witnessed, that he sent him a present of twenty rupees. I hear the other man is going on favourably. I saw them both in the Station Hospital at Mian-Nu on Friday, and they were then cheerful, and thought to be going on all right.

The Punjab chiefs were all encamped on the ground by the fort; we drove down through the city to see them; their lamps were most picturesque—coloured bunting, tents, flags, Chinese lanterns, and fairy lamps. We enjoyed our drive back through the city, buying many pretty things. In the evening there was a grand ball, the entrance into the gay world of many a young *débutante*, and 1,000 people were present.

Then came the day of the durbar, and we were up betimes and off to the Viceroy's camp, having good seats close to the entrance to the Viceroy's tent, where the durbar was held. As each rajah arrived in his state carriage with his guard of honour, guns fired salutes according to rank (nineteen being the most). Patiola's strings of pearls, as large as hazel nuts, were much admired; they are the finest of his jewels, though his turban was a mass of diamonds. His brother's turban was festooned with strings of diamonds. Karputhala's turn-out was the next best. There were two silver carriages, and by three of the rajahs—state umbrellas were carried. A rajah from Beloochistan wore long hair and had a crown of diamonds on his head. The durbar tent, when they were all assembled, presented a grand appearance; the body-guard all stood back to back down the centre, holding their lances. The rajahs, eleven in number, and their followers, sat down each side facing them. The Viceroy was on a daïs covered with crimson and cloth of gold, and five officers from each regiment were present, seventeen generals, and many civilians of note, the Lieut.-Governors of the Punjab and North-West Provinces, the Governor of Bombay, and twenty ladies. The speeches have already appeared in the English papers. After the durbar, the rajahs all departed in state in the same rank as they came. Although the state howdah used by the Viceroys at previous durbars is still extant, the state elephants have all died of anthrax, and not been replaced; the trappings of the howdah are very gorgeous. The durbar tent used for the Viceroy's reception was a vast hall, beautifully carpeted, and everyone revolved round the host like planets round the sun, no one being expected to turn his or her back, or sit down in his presence. We rubbed elbows with rajahs and the grandest dignitaries in the land, everyone being in full dress. One rajah had a handsome coat, with a fleur-de-lis, about four inches long and three broad, composed of diamonds, on each breast. Many of the rajahs and other native gentlemen spoke English perfectly. The Viceroy's drawing-room was beautifully furnished, and it was well-nigh impossible to realise that they were tents, for they had proper fire-places and glass windows. The extremely neat, though striking, uniform of two quiet and lady-like nursing sisters was much admired by many present. Some of the troops moved on from the durbar to Wagiristan, and it is said that orders came for the Gurrkhas to march, and the very next morning, when the medical officer visited the Gurrkha Hospital, he found it empty, for the sick had deserted their beds at the prospect of war.

### Notes and Queries.

### Queries.

(131) *Training.*—I am advised to ask your assistance with regard to obtaining hospital training. I am 22, and wish to fit myself for medical work in foreign mission fields.—*A. M. B.*

### Answers.

(131) *Training* (*A. M. B.*)—From your letter you seem to confuse medical with nursing work. For the former, you must go through five years' preparation to qualify you for practising at home or abroad. You can get particulars from the secretary of the School of Medicine for Women, Handel Street, London, W.C., or read "How to Become a Medical Woman" in The Hospital, of September 10th, and September 17th, 1892. For training as a nurse, three years are required at many hospitals, but you could not begin till 24 or 25. Some infirmaries and provincial hospitals accept candidates at 23. You would get good training at a workhouse infirmary where the superintendent of nurses is a properly trained nurse.

### Where to Go.

Athenæum, Camden Town.—A bazaar in aid of the North-West London Hospital will be opened on May 13th by the Marquis of Camden.

A Preparation Class for the London Obstetrical Society's July examination is now being arranged by the Secretary, Midwives' Club, 12, Buckingham Street, Strand, London. Classes for massage are held at the same address under the auspices of the Society of Trained Masseuses.

Grosvenor House.—On May 11th, Her Royal Highness Princess Christian has promised to attend the annual distribution of certificates and medals by the National Health Society. The meeting will be held at Grosvenor House by the kind permission of the Duke of Westminster.

H.R.H. the Duchess of York has selected the Victoria Hospital for Children as the institution which she wishes should benefit by a grand evening concert, to be given by the Royal Amateur Orchestral Society, at Queen's Hall, on May 18th, and at which their Royal and Imperial Highnesses the Duke and Duchess of Saxe-Coburg Gotha, and their Royal Highnesses the Duke and Duchess of York will be present.

The New Gallery—Summer Exhibition.—The private view at the New Gallery was held on Saturday, and the first of the summer shows now opens its doors to the public. Of this exhibition, as a whole, perhaps one can barely say that it is above the average, though certain very notable exceptions among the 400 works exhibited raise it out of any ordinary level, and will make it in all likelihood the gallery of the season. And it is of the exceptions, the pictures which most prominently stand out, we intend to speak now. Take Burne-Jones, for instance, who is represented this year not only by the quality but by the quantity of his works. Each of these pictures is remarkable, and two, at least, show the master at his best. Vis-à-vis to one another in the West Room are his two principal canvases, "The Sleeping Beauty" (No. 106) and "The Marriage of Psyche" (No. 163), the former of these being an early design of the fourth study of the Brier Rose series. The quiet and calm of the picture are not its least attractions. The hurry and bustle of life are half forgotten as one looks, such suggestiveness of rest are depicted by the recumbent figures. Sleep—utter rest and peace, pervades a canvas painted in exquisite harmony with the subject. Of the same peculiar charm is the artist's other central canvas, and in "The Marriage of Psyche," Burne-Jones is equally at his best. A procession of maidens, preceding the fair goddess, forms the scheme of the picture. Demure, simple, and unconscious, Psyche's companions are in curious contrast in their maidenly dignity to the pervading ideal of the womanhood of 1895. It has been written of this picture that it is too melancholy a representation of a wedding procession, but surely for "melancholy" one should read thoughtful, and it has certainly little in common with more modern marriage groups, as exemplified to us nowadays. In the North Room, Mr. Sargent's portrait of Miss Ada Rehan holds a certain sovereignty. Here we are shown the woman, not the actress, painted in vivid lifelike colouring. This canvas is at once a picture and a portrait, and Mr. Sargent shows himself at his strongest in the present instance of his work. Mr. Shannon has some good work also, but his "Kit" (No. 113) remains a mystery to us. A mist—in other words a smudge—seems to obscure the child's whole person, but whether this is an experiment in advanced impressionism, or an unforseen accident, we are not told. An able study in artificial light is shown in John Collier's picture (No. 238) "The Laboratory." The picture is a remarkable one as a study of artificial light treated in an unartificial manner. Of landscape work there is much that is striking, a little that is bad, in the exhibition. The central position in the North Room is occupied by Mr. Frank East's "A Misty Mere," a picture painted in a very different tone of colour to the one hanging there last season, by the same painter; the present one is less striking, less individual. "The Avenue by the Marshes," by Mr. Aaron Stokes, is prhaps the finest and most pleasing specimen of landscape art this year.

### Everybody's Opinion.

[Correspondence on all subjects is invited, but we cannot in any way be responsible for the opinions expressed by our correspondents. No communications can be entertained if the name and address of the correspondent is not given, or unless one side of the paper only be written on.]

### A HINT TO CANDIDATES.

"THE SECRETARY OF A PROVINCIAL INFIRMARY" writes: Having had experience in reviewing applications for the appointment of matron, I should like to give a few hints to aspirants for such appointments as to the manner in which they should frame their applications. The handwriting should be legible and suitable paper used. I am confident that many applications, perhaps from well qualified women, are thrown on one side owing to the faulty style of manuscript. The applicant should state age and record her services seriatim and concisely, thus:—" Served............Hospital from......... to.........received certificates for.........at.........Hospital from .........to.........as............" and so on. Then add any special qualifications. Certificates of nursing should not be sent, nor original testimonials; printed copies are much preferable to manuscript; one or two letters as to social position are desirable. References are of little use, as a committee of selection will not trouble to refer, they judge from the papers submitted. The applicant's signature should be legible as also the address, the Christian name signed in full—in fact, a woman should invariably sign her Christian name in full, so as to avoid mistake in the sex when addressing a reply.

### PRIVATE NURSING.

SISTER LUCIE, of the Enfield Nursing Association, writes: Having read various letters on private nursing contributed to THE HOSPITAL, I beg leave to say a few words on the matter. As a nurse of nearly seventeen years' standing, I have been sister and night superintendent in hospitals, and have done a fair share of private nursing at home and abroad. It seems to me that a most important fact has been lost sight of, viz.: It entirely depends on the patient's complaint whether the nurse in charge of the case can leave the sick-room or not. In typhoid or a major operation, or in an unconscious or delirious case, constant watching and careful feeding at regular intervals are required. Would it, therefore, be just to the patient, his relatives, or the doctor, for the nurse to go down to the kitchen to help cook the family dinner, or to do any self-imposed task? Surely the nurse's place is in the sick-room, which she ought to dust and keep pure, emptying all secretions. As a rule, people do not call in trained nurses, except in grave sickness, for their fees are too high for many pockets, and nearly always there is some kind friend who can see to the housekeeping, if the nurse restrains her attention to the sick-room and its duties. Fatal leaps from windows are always taken by a delirious patient in the absence of the attendant whose duty it was to watch the case. If some unwise relative be left with a typhoid, food is often given which may cost a life. If an operation case is left alone, or in charge of someone who moves a bandage or helps the patient to ease a position, the nurse may find on her return from the kitchen stitches broken down or hemorrhage going on unnoticed. A nurse must, of course, leave the sick-room to eat her food, take exercise, or get rest, but she endeavours to secure a reliable substitute. Patients should remember that a private nurse goes on from year's end to year's end, from case to case, and requires regular meals, outdoor exercise, and proper rest either by day or night. These are necessary in order that good nursing may be insured for the patient, as an overworked woman cannot efficiently do her duty.

[It is not wise for employers to ask trained nurses to take their meals in the kitchen. Whether they are gentlewomen or not, it is not fair to require them to do what is never demanded of the governess, companion, or mother's help.—ED. T. H.]

### Registration of Midwives.

WE have received the following from Mr. J. H. Rutherglen, clerk to the Kensington Guardians:—

The Guardians of this parish, who for some years past have trained in the special lying-in wards attached to their infirmary a large number of trained nurses to pass the examination of the London Obstetrical Society, have heard with much regret of the resolution passed by the General Medical Council on December 3rd last with respect to the issue of midwifery diplomas or certificates in general, and of the certificates of the Obstetrical Society of London in particular.

The Guardians are the more surprised at this action as they have always understood that the General Medical Council have regarded the absence of public provision for the education and supervision of midwives as being productive of a large amount of suffering and disease among the poorer classes, and that the Council had been recommended by a Select Committee of the House of Commons to frame rules for the conduct of examinations for the admission of women to act as trained and certificated midwives.

It was to a great extent to meet these expressions of opinion that the Guardians of this parish undertook, in conjunction with the Workhouse Infirmary Nursing Association, the yearly training of a number of previously qualified nurses in their midwifery wards, but they have every reason to fear that if the resolution of the Council stands, and the London Obstetrical Society therefore ceases to issue in its present form its diploma or certificate of competency, the good work which they have been doing for so many years will cease as women will be unwilling to enter for a course of training for which they will be unable to obtain a certificate of efficiency qualifying them to act as midwives.

Having regard, therefore, to the value and importance which is attached to a special training in midwifery, and to the examinations conducted by the London Obstetrical Society and other bodies, the Guardians have directed me to ask that the General Council will reconsider and withdraw their resolution of December last, so that the training and sending out of competent midwives may be continued unimpeded.

Bearing on the above subject, we have received a communication from the secretary of the Lay Association for Promoting the Compulsory Registration of Midwives, desiring to draw attention of the women of England to the great need for legislation in the matter of midwives. It is therein stated that

Any woman, however incapable, and absolutely untrained, can at present call herself a midwife, and have a plate with the word midwife upon it on her door, and no one can prevent her! The amount of misery and suffering caused by this untrained attendance is inconceivable! It is not usually realised how very general amongst women of the poorer classes is the custom of employing midwives; in some parts of England even to the extent of 90 per cent. and over. Statistics show that among working women at least seven out of ten births take place without a medical man! A Select Committee of the House of Commons sat and took evidence as to the practice and result of midwifery by midwives in Great Britain, and reported that legislation was imperative! . . . It is in no sense intended to create a new class of practitioners, but we trust it will be the means, by making compulsory a short, but thorough training for midwives in the management of normal cases (the only cases that a trained and registered midwife is entitled to undertake), of protecting our poorer sisters from becoming the victims of ignorance. . . . Registered midwives exist in every country in Europe but England, and are proved to work satisfactorily. We plead especially with the women of England to interest themselves in this subject.

### Appointments.

PLYMOUTH WORKHOUSE HOSPITAL.—Miss Alice Maud M. Tilbury has been appointed Superintendent of Nurses at this workhouse hospital. She was trained at the London Hospital, where she afterwards held the post of staff nurse. For the last two years Miss Tilbury has been matron's assistant and superintendent of nursing at Lambeth Infirmary. Her testimonials are good, and we wish her every success in her new work.

### The Book World for Women and Nurses.

[We invite Correspondence, Criticism, Enquiries, and Notes on Books likely to interest Women and Nurses. Address, Editor, The Hospital (Nurses' Book World), 428, Strand, W.C.]

First Aid to the Injured and Management of the Sick. By E. J. Lawless, M.D., D.P.H. (Edinburgh and London: Young J. Pentland.)

This book will prove extremely useful to many besides the volunteer bearers for whom it is primarily intended, and the treatment and warnings given respecting fractures are of universal application. Arrest of hæmorrhage, treatment of frost bite, poisoned and other wounds, are detailed in simple vigorous language which adds to the attractions of this capitally illustrated volume. The general health of the volunteer, his clothing bathing, &c., receive due attention from Dr. Lawless, and his advice on many points might well be also followed by civilians. To the paragraph on enemas exception must be taken, for the difference between nutrient and aperient injections not being defined, the reader is left with an impression that Higginson's syringe is recommended for both, whilst no mention is made of the quantity of fluid usually ordered in either case.

Manual for the Church Lads' Brigade Medical Staff Corps. (Published at the Church House, Dean's Yard, Westminster. Price 4d.)

This very small book contains a vast amount of information, besides two diagrams of the skeleton and the arteries. It has been compiled and edited by Dr. Prosser White, Surgeon-Major 1st (Wigan) Battalion Liverpool Regiment. The drill and course of instruction are taken from the "Manual for the Army Medical Staff Corps." The popularity of this pamphlet is not likely to be confined to members of the Church Lads' Brigade.

Infancy and Infant Rearing. By John Benjamin Hellier, M.D.Lond., M.R.C.P.Eng. (Published by Charles Griffin and Co., Exeter Street, Strand.)

Nearly thirty illustrations embellish Dr. Hellier's latest work, which is specially prepared for "pupil midwives and other nurses who seek a scientific understanding of their work." Interesting statistics on infant mortality are followed by instructions on child rearing, which should, if carried out, considerably lower the death rate. In the chapter on "The General Hygiene of Infancy" no sentence exceeds in practical value the one treating of clothing, where we read, "Keep the rooms from becoming too hot; keep the child from becoming too cold."

The Rearing and Feeding of Children. By Thomas Dutton, M.D., Univ., Durh. (Henry Kimpton, 82 High Holborn, and Hirschfeld Bros., Bream's Buildings, Fetter Lane. Price 2s.)

The author of this mother's guide explains in his preface that he is "no advocate, generally speaking, for the amateur doctoring of children," but "when medical aid is not obtainable the mother can do a great deal with the help of a little knowledge to ward off disease." It is with the object of supplying some of this needful knowledge that the present prettily bound and admirably printed volume has been placed before the public, and the advice on the early care of infants is excellent. So are the warnings against improper foods, soothing syrups, &c. The position of the nursery and even the chairs suited to growing children are indicated, for the author wisely condemns the straight-back, cushionless furniture on which former generations did penance. Dr. Dutton is strongly in favour of nursemaids being properly trained, and advocates the employment of ladies as children's nurses, a plan which he considers likely to be mutually beneficial. The article on "Sleep" is one of the best in the book, and might be advantageously studied by all who have the care of young people. In the chapter devoted to slight ailments, the author will probably, in a second edition, see the propriety of somewhat elaborating his instructions for the benefit of inexperienced readers. A dictionary may enlighten the latter as to the meaning of words such as "prophylactic," but in recommending such delicate operations as the syringing of nose or eye, it would be surely well to add a warning as to needful precautions. The victim to "dirt and gravel" in the eye might have his sufferings considerably increased by the vigorous cold syringing of an amateur.

### The Volunteer Medical Staff Corps at Netley.

By One of the Party.

It had been originally intended that the London companies of the Volunteer Medical Staff Corps proceeding to the Royal Victoria Hospital at Netley on Easter Thursday for their annual course of training should form a camp in the hospital grounds, but this order was at the last moment countermanded, room for housing the men being found partly in the main building and partly in barracks in the rear. A good muster of the corps paraded at Waterloo Station under command of Surgeon Lieut.-Colonel A. T. Norton, V.D., Surgeon Captain and Adjutant D. M. O'Callaghan, A.M.S. and reached Netley Hospital soon after ten o'clock. During the visit the orders for the day were generally: Reveille at half-past five a.m., first parade at a quarter-past six, for company or battalion drill; breakfast at a quarter-past eight; second parade at a quarter-past nine, for stretcher and field work; dinner at one p.m.; tea at five; and "lights out" at ten p.m. Meals were served in the Garrison Theatre; suppers could be ordered after seven p.m. in the "dry" canteen. As a rule, after dinner the men were at liberty to go where they pleased, with the exception of those warned for guard, fire picket, "fatigue," or other duties. The grounds and jetty afford an excellent promenade for convalescents the foreshore along the front is, by hospital rules, placed out of bounds for the patients. The sick and wounded are not landed at this jetty, but discharged from the transports at Portsmouth and conveyed by rail to the Government siding at Netley. A party, collected from Jamaica, Barbadoes, the Bermudas, and other West Indian stations, came in on Good Friday. How weary most of the poor fellows seemed! One other batch of sick will come from the East, and then the trooping season will end. Numbers of young surgeons on probation are stationed here; also a large staff of nursing sisters in their picturesque scarlet capes and grey dresses. A route march was ordered for Good Friday, and on Saturday a lecture on "Organisation" was delivered to the corps by Brigade Surgeon Lieut.-Colonel G. J. H. Evatt, who referred to the shame brought on the nation by the condition of the base hospital at Scutari, where one woman's masterly mind evolved order out of chaos. He spoke of the subsequent formation of the Army Hospital Corps, its duties in the field, of hospital-ships and sick transports, and of the recent reforms in the formation of brigade bearer companies. The block in the base hospitals at Ismalia during the campaign of 1882 was, the lecturer said, due (though aggravated by the absence of proper drainage and water supply) to the disproportion of the medical and nursing staffs to the numbers of sick and wounded, and he urged the necessity of largely increasing the Medical Staff Corps. On Monday the lecture was practically illustrated in the grounds of the hospital, the course of the wounded soldier being traced from the fighting line to the base at "Netley," further instruction being given at each station of medical relief. In the afternoon a football match was arranged between the M.S.C. Football Club and a team from the V.M.S.C., and on Sunday church parade was held in the garrison chapel. A march past and an inspection by the Principal Medical Officer brought an instructive and pleasant visit to a close.

## Figures and Tables

**Fig. 23 f1:**
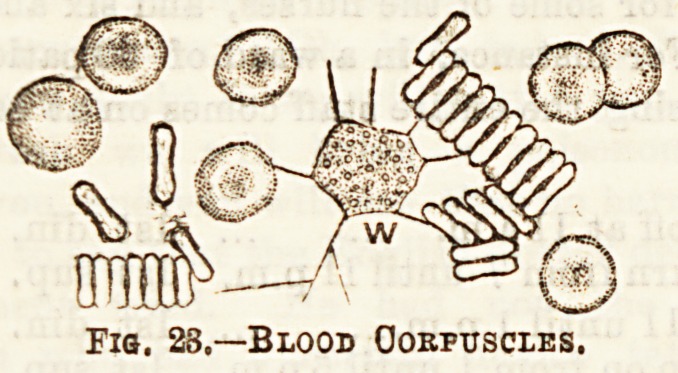


**Fig. 24 f2:**
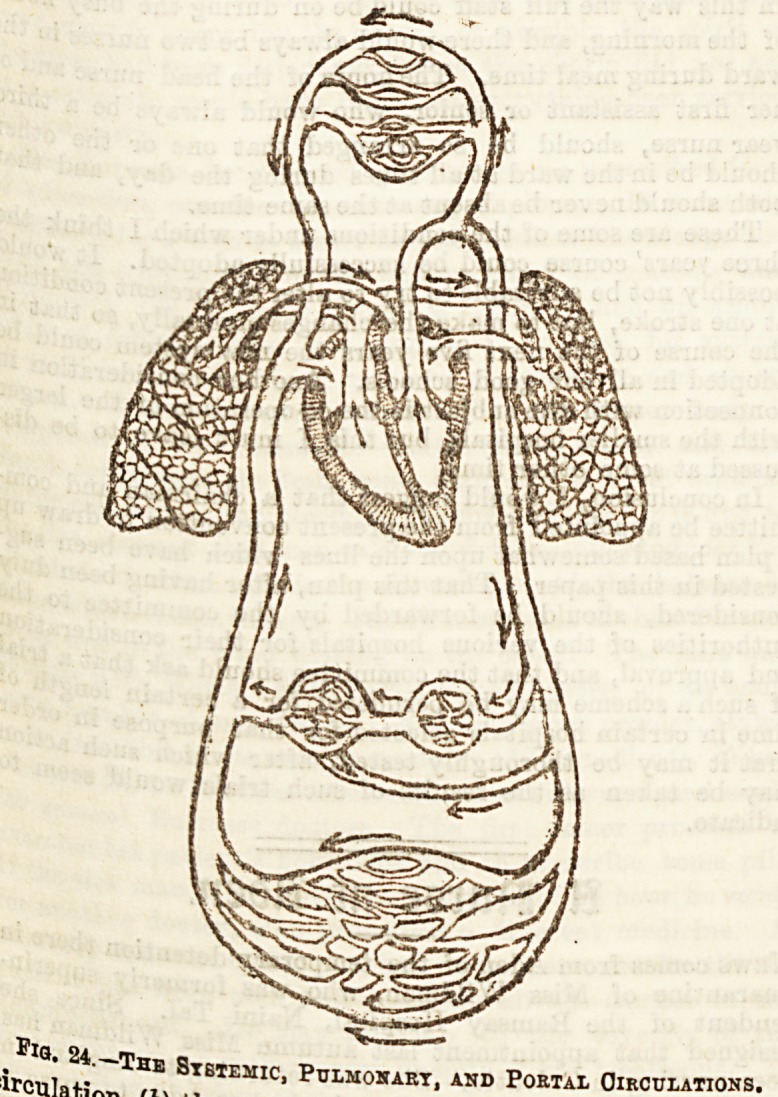


**Fig. 25 f3:**
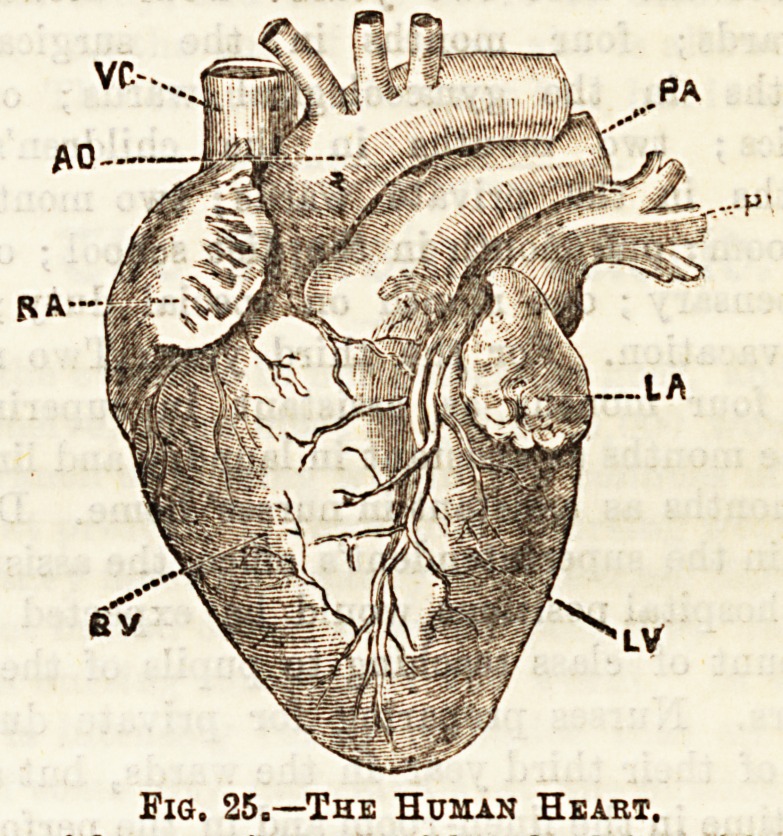


**Figure f4:**